# Supplementary figures and images for: Relationships between fox populations and rabies virus spread in northern Canada
Source: PLoS One. 2021 Feb 16;16(2):e0246508. doi: 10.1371/journal.pone.0246508 (PMC7886166; doi:10.1371/journal.pone.0246508)

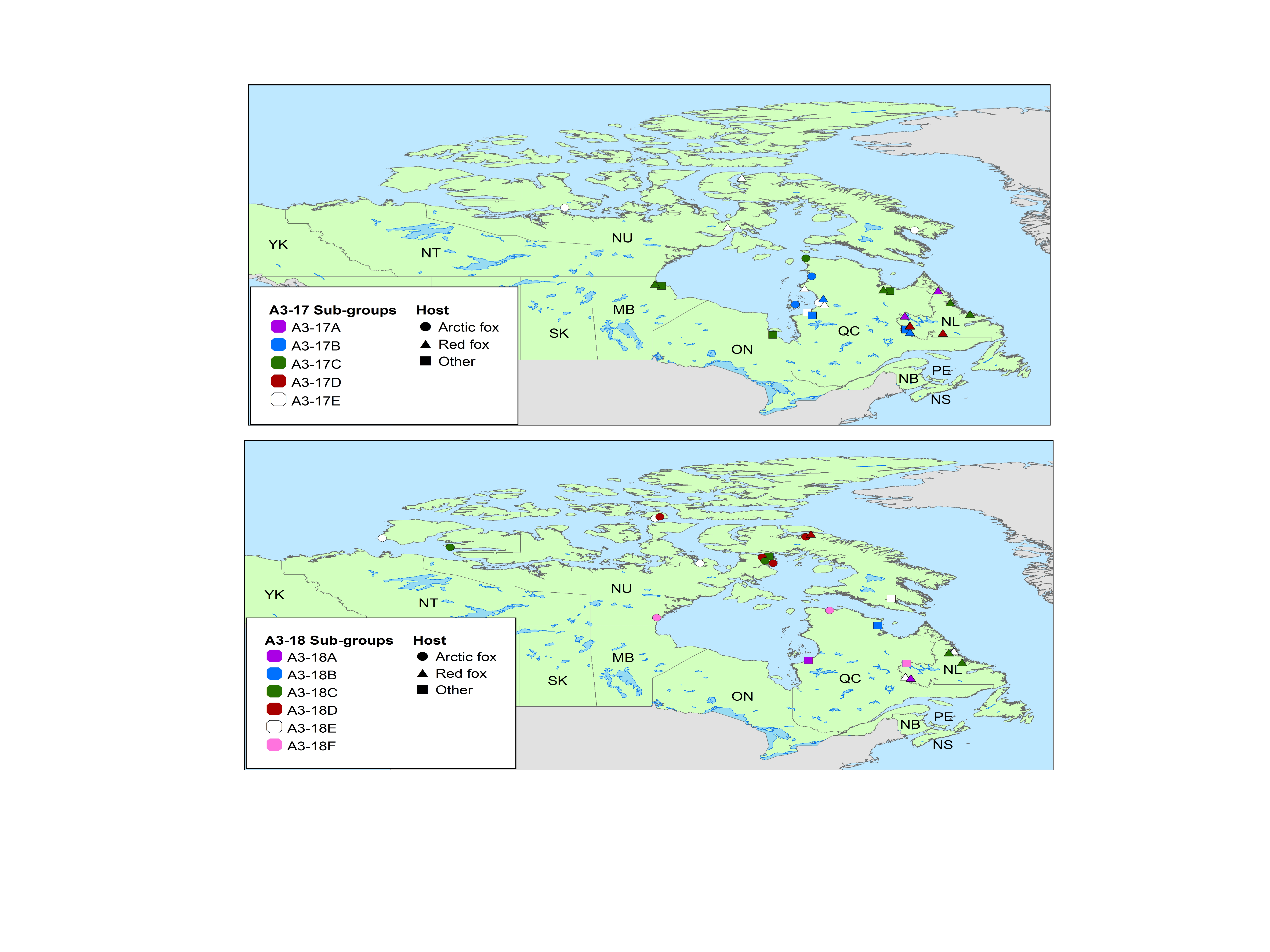

Supplement: S1 Fig — The maps were generated as described for Fig 4. (PNG) [file pone.0246508.s001.png]

## Slide 1
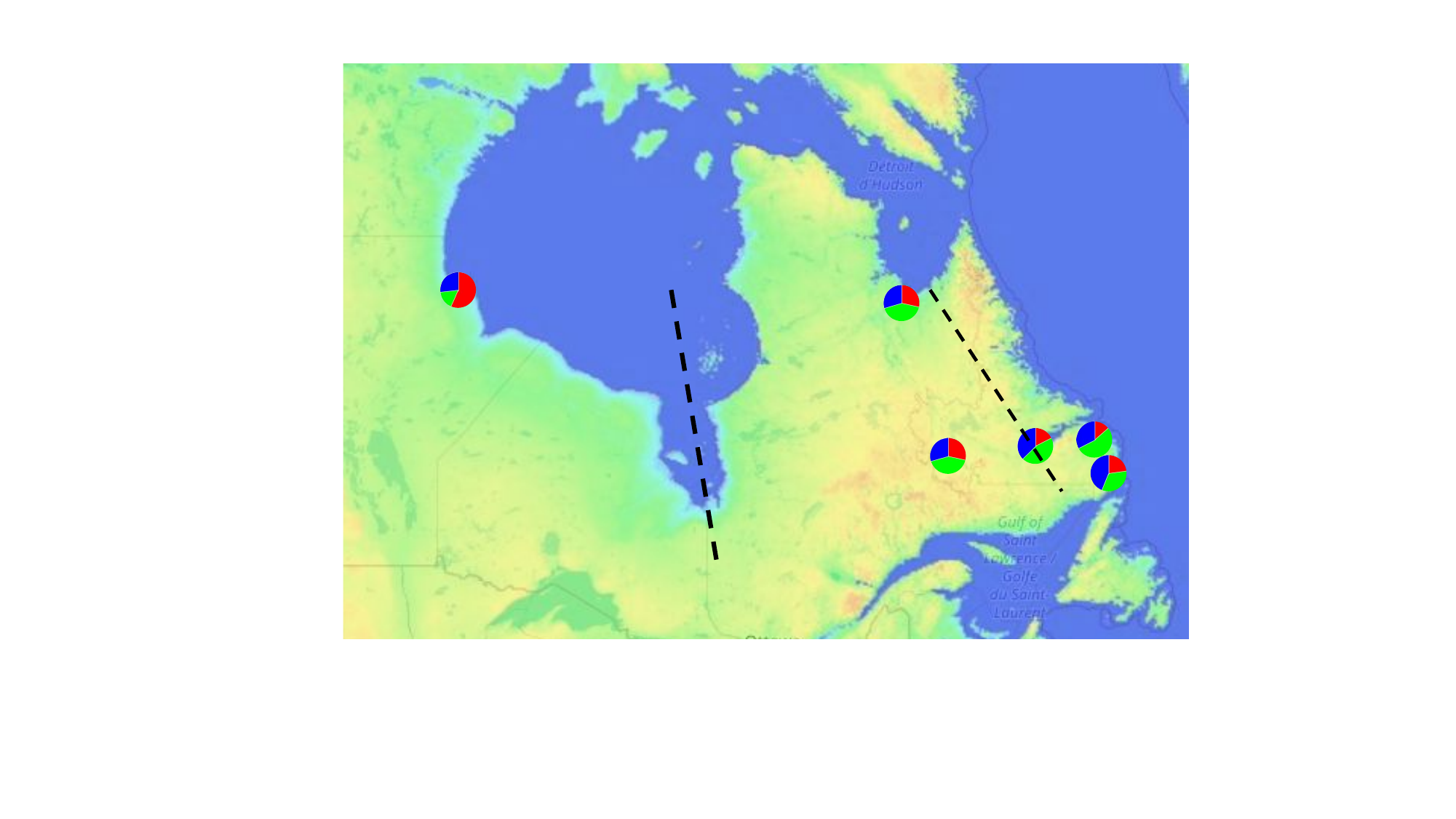

Supplement: S2 Fig — The pie charts of each genetic cluster are as presented in Fig 7A. Dashed lines indicate changes in pattern of genetic variation between northwestern vs northeastern localities (left) and interior and coastal localities (right). The map was generated in topographical-map.com. (PPTX) [file pone.0246508.s002.pptx]
