# Supplementary material for: Relationships between fox populations and rabies virus spread in northern Canada
Source: PLoS One. 2021 Feb 16;16(2):e0246508. doi: 10.1371/journal.pone.0246508 (PMC7886166; doi:10.1371/journal.pone.0246508)
Supplement: S2 Table — (DOCX) [file pone.0246508.s004.docx]

S2 Table. Characteristics of sites identified by MEME as being under positive selection.

| Site in concatenated ORF | Protein location | Amino acid substitution | Sub-lineage and group members affected | No. of isolates affected | P-value |
| --- | --- | --- | --- | --- | --- |
| 3139 | L1666 | Q to K | A3-2,7,14 | 9 | 0.01 |
| 863 | M116 | L to S | A3-5,18 | 2 | 0.03 |
| 2800 | L1327 | I to V or L | A3-14,17,18 | 3 | 0.04 |
| 14 | N14 | V to I | A4 | 2 | 0.05 |
| 3517 | L2044 | R to Q | A4, A3-17, A1-ON4 | 6 | 0.06 |
| 774 | M27 | D to N | A3-8 | 1 | 0.07 |
| 612 | P162 | S to F or P | A3-12,17 | 3 | 0.09 |
| 1436 | G487 | K to E or M | A3-3,9 | 2 | 0.09 |
| 1520 | L46 | A to T or S | A3-4,18 | 2 | 0.09 |
