# Supplementary material for: Relationships between fox populations and rabies virus spread in northern Canada
Source: PLoS One. 2021 Feb 16;16(2):e0246508. doi: 10.1371/journal.pone.0246508 (PMC7886166; doi:10.1371/journal.pone.0246508)
Supplement: S3 Table — (DOCX) [file pone.0246508.s005.docx]

S3 Table. Occurrence and distribution of 18 control region haplotypes among 162 red foxes, including 23 rabies positive animals, across Canada. Haplotypes are designated r1-18 according to their frequency of occurrence in this study; information indicates designation in other studies (Langille et al. 2014; Aubry et al. 2009; Statham et al. 2012). Locality codes are given in Table 1. Other refers to the other localities listed in Table 1. RP is the number of rabies-positive foxes with the haplotype.

| Haplotype | Information | CAR | CHV | KUU | LAB | NWR | PHS | Other | Total | RP |
| --- | --- | --- | --- | --- | --- | --- | --- | --- | --- | --- |
| r1 | NL1=Aubry9 | 9 | 8 | 9 | 10 | 13 | 9 | 3 | 61 | 6 |
| r2 | NL2=Aubry17 | 9 | 5 | 4 | 6 | 7 | 3 | 3 | 37 | 8 |
| r3 | NL3 | 0 | 14 | 0 | 2 | 0 | 1 | 0 | 17 | 3 |
| r4 | NL6=Aubry79 | 0 | 4 | 2 | 4 | 3 | 2 | 2 | 17 | 1 |
| r5 | Aubry24 | 0 | 8 | 0 | 0 | 0 | 0 | 0 | 8 | 0 |
| r6 | Aubry63 | 0 | 3 | 0 | 0 | 0 | 0 | 0 | 3 | 0 |
| r7 | Aubry73 | 0 | 1 | 0 | 0 | 0 | 1 | 1 | 3 | 1 |
| r8 | Statham87 | 0 | 3 | 0 | 0 | 0 | 0 | 0 | 3 |  |
| r9 | Aubry60 | 0 | 2 | 0 | 0 | 0 | 0 | 0 | 2 |  |
| r10 | New | 0 | 0 | 0 | 0 | 1 | 1 | 0 | 2 |  |
| r11 | New | 0 | 0 | 0 | 0 | 0 | 0 | 2 | 2 | 2 |
| r12 | Aubry59 | 0 | 0 | 0 | 0 | 0 | 0 | 1 | 1 | 1 |
| r13 | Aubry61 | 0 | 0 | 0 | 0 | 0 | 0 | 1 | 1 | 1 |
| r14 | Aubry7 | 0 | 1 | 0 | 0 | 0 | 0 | 0 | 1 |  |
| r15 | Aubry75 | 0 | 0 | 0 | 0 | 0 | 0 | 0 | 1 |  |
| r16 | New | 0 | 0 | 0 | 0 | 0 | 1 | 0 | 1 |  |
| r17 | Statham26 | 0 | 1 | 0 | 0 | 0 | 0 | 0 | 1 |  |
| r18 | New | 0 | 0 | 1 | 0 | 0 | 0 | 0 | 1 |  |
| Total |  | 18 | 50 | 16 | 22 | 24 | 18 | 13 | 162 | 23 |
