# Supplementary material for: Relationships between fox populations and rabies virus spread in northern Canada
Source: PLoS One. 2021 Feb 16;16(2):e0246508. doi: 10.1371/journal.pone.0246508 (PMC7886166; doi:10.1371/journal.pone.0246508)
Supplement: S4 Table — (DOCX) [file pone.0246508.s006.docx]

S4 Table. Occurrence and distribution of 33 control region haplotypes among 157 arctic foxes, including 27 rabies positive animals, across Canada. Haplotypes are designated a1-31 according to their frequency of occurrence in this study. Information refers to haplotype designation in Dalen et al. (2004). Locality codes are given in Table 1. Other refers to the other localities listed in Table 1. RP is the number of rabies-positive foxes with the haplotype.

| Haplotype | Information | BAK | IGL | CHV | RAG | RAN | SAC | Other | Total | RP |
| --- | --- | --- | --- | --- | --- | --- | --- | --- | --- | --- |
| a1 | H1 | 3 | 5 | 18 | 3 | 12 | 3 | 6 | 50 | 10 |
| a2 | H9 | 2 | 3 | 7 | 0 | 1 | 0 | 1 | 14 | 1 |
| a3 | H8 | 2 | 2 | 6 | 0 | 0 | 1 | 0 | 11 | 3 |
| a4 | H7 | 0 | 0 | 6 | 2 | 0 | 0 | 1 | 9 | 1 |
| a5 | H2 | 2 | 0 | 3 | 2 | 1 | 0 | 1 | 9 | 1 |
| a6 | N1 | 2 | 0 | 2 | 0 | 0 | 1 | 3 | 8 | 3 |
| a7 | S2 | 1 | 1 | 1 | 4 | 0 | 0 | 0 | 7 |  |
| a8 | H5 | 0 | 1 | 3 | 0 | 0 | 0 | 2 | 6 | 2 |
| a9 | C3 | 1 | 3 | 1 | 0 | 0 | 0 | 0 | 5 |  |
| a10 | H4 | 1 | 0 | 0 | 3 | 0 | 0 | 1 | 5 | 1 |
| a11 | C4 | 1 | 0 | 2 | 0 | 0 | 0 | 1 | 4 | 1 |
| a12 | G2 | 1 | 0 | 0 | 0 | 1 | 1 | 0 | 3 | 1 |
| a13 | H6 | 0 | 1 | 2 | 0 | 0 | 0 | 0 | 3 |  |
| a14 | New | 0 | 1 | 1 | 0 | 0 | 0 | 0 | 2 |  |
| a15 | N3 | 0 | 1 | 0 | 0 | 0 | 0 | 1 | 2 | 1 |
| a16 | C2 | 0 | 0 | 1 | 0 | 0 | 0 | 1 | 2 |  |
| a17 | New | 0 | 1 | 0 | 0 | 0 | 0 | 0 | 1 |  |
| a18 | New | 0 | 1 | 0 | 0 | 0 | 0 | 0 | 1 |  |
| a19 | New | 0 | 1 | 0 | 0 | 0 | 0 | 0 | 1 |  |
| a20 | New | 0 | 1 | 0 | 0 | 0 | 0 | 0 | 1 |  |
| a21 | New | 0 | 1 | 0 | 0 | 0 | 0 | 0 | 1 |  |
| a22 | New | 0 | 0 | 0 | 0 | 1 | 0 | 0 | 1 |  |
| a23 | C1 | 0 | 0 | 1 | 0 | 0 | 0 | 0 | 1 |  |
| a24 | New | 0 | 0 | 1 | 0 | 0 | 0 | 0 | 1 |  |
|  |  |  |  |  |  |  |  |  |  |  |
| a25 | G3 | 0 | 0 | 1 | 0 | 0 | 0 | 0 | 1 |  |
| a26 | New | 0 | 0 | 1 | 0 | 0 | 0 | 0 | 1 |  |
| a27 | New | 0 | 0 | 1 | 0 | 0 | 0 | 0 | 1 |  |
| a28 | New | 1 | 0 | 0 | 0 | 0 | 0 | 0 | 1 |  |
| a29 | C6 | 0 | 0 | 0 | 0 | 0 | 1 | 0 | 1 | 1 |
| a30 | New | 0 | 0 | 0 | 0 | 0 | 0 | 1 | 1 | 1 |
| a31 | New | 0 | 1 | 0 | 0 | 0 | 0 | 0 | 1 |  |
| a32 | New | 0 | 0 | 0 | 1 | 0 | 0 | 0 | 1 | 0 |
| a33 | New | 0 | 0 | 0 | 1 | 0 | 0 | 0 | 1 | 0 |
| Total |  | 17 | 24 | 58 | 16 | 16 | 7 | 19 | 157 | 27 |
