# Supplementary material for: Relationships between fox populations and rabies virus spread in northern Canada
Source: PLoS One. 2021 Feb 16;16(2):e0246508. doi: 10.1371/journal.pone.0246508 (PMC7886166; doi:10.1371/journal.pone.0246508)
Supplement: S5 Table — (DOCX) [file pone.0246508.s007.docx]

S5 Table. Characteristics of microsatellite loci used in this study. For F-statistics, numbers in bold are significant at P=0.05 after Bonferroni correction for multiple tests. *N*_A_ = number of alleles; *H*_E_ = expected heterozygosity.

| Locus | Allelic Range | *N*_A_ | *H*_E_ | *F*_IS_ | *F*_ST_ |
| --- | --- | --- | --- | --- | --- |
| *Vulpes vulpes* | | | | | |
| CO4.140 | 14 | 8 | 0.666 | 0.0376 | 0.0317 |
| AHTh171 | 28 | 6 | 0.409 | -0.0298 | **0.0572** |
| REN105L03 | 16 | 9 | 0.827 | -0.0629 | **0.0372** |
| CPH3 | 22 | 9 | 0.635 | 0.1392 | 0.0199 |
| AHT121 | 30 | 13 | 0.826 | 0.0072 | 0.0202 |
| REN247M23 | 16 | 8 | 0.687 | **0.1955** | 0.0203 |
| CPH9 | 10 | 5 | 0.635 | -0.0201 | **0.0380** |
| CPH15 | 16 | 9 | 0.790 | -0.0622 | 0.0250 |
| CO1.424 | 14 | 7 | 0.600 | -0.0245 | **0.0639** |
| Mean | 18.4 | 8.22 | 0.675 | 0.0200 | 0.0348 |
| *Vulpes lagopus* |  |  |  |  |  |
| CO4.140 | 22 | 12 | 0.836 | -0.0102 | 0.0128 |
| AHTh171 | 30 | 13 | 0.838 | -0.0022 | 0.0120 |
| REN105L03 | 22 | 11 | 0.843 | **0.1154** | -0.0016 |
| CPH3 | 32 | 17 | 0.902 | 0.0120 | 0.0112 |
| AHT121 | 38 | 16 | 0.876 | -0.0722 | 0.0033 |
| REN247M23 | 26 | 11 | 0.805 | 0.0463 | 0.0182 |
| CPH9 | 14 | 7 | 0.673 | 0.0464 | -0.0028 |
| CPH15 | 20 | 10 | 0.581 | 0.0158 | 0.0037 |
| CO1.424 | 28 | 11 | 0.598 | 0.0252 | 0.0341 |
| Mean | 25.8 | 12 | 0.772 | 0.0196 | 0.0101 |
