# Supplementary material for: Relationships between fox populations and rabies virus spread in northern Canada
Source: PLoS One. 2021 Feb 16;16(2):e0246508. doi: 10.1371/journal.pone.0246508 (PMC7886166; doi:10.1371/journal.pone.0246508)
Supplement: S6 Table — (DOCX) [file pone.0246508.s008.docx]

S6 Table. Mitochondrial control region haplotypes of the 50 rabies-positives foxes collected from different parts of Canada during the 2012-2013 rabies outbreak.

| Sample ID | Mitochondrial Haplotype | Microsatellite Cluster | Viral group | Location |
| --- | --- | --- | --- | --- |
| **Red Foxes** (n=23) |  |  |  |  |
| QC.2012.0988 | r11 | 3 | 17 | Inukjuak QC |
| NL.2012.1054 | r11 | 3 | 17 | Nain NL |
| QC.2012.1351 | r1 | 3 | 17 | Kuujjuaq QC |
| NL.2012.0627 | r2 | 3 | 18 | Wabush NL |
| NL.2012.0579 | r1 | 3 | 18 | Wabush NL |
| NL.2012.0580 | r3 | 3 | 17 | Labrador City NL |
| NL.2012.0672 | r2 | 3 | 17 | Hebron NL |
| NL.2012.0628 | r1 | 3 | 18 | Wabush NL |
| NL.2012.0846 | r2 | 3 | 18 | Nain NL |
| QC.2012.0847 | r1 | 1 | 17 | Fermont QC |
| NL.2012.0857 | r7 | 3 | 17 | Makkovik NL |
| NL.2012.0449 | r2 | 3 | 18 | Wabush NL |
| NL.2012.0450 | r4 | 3 | 18 | Wabush NL |
| NL.2012.0130 | r1 | 3 | 17 | Wabush NL |
| NL.2012.0215 | r2 | 3 | 18 | Wabush NL |
| QC.2012.0247 | r2 | 1 | 17 | Schefferville QC |
| NL.2012.0279 | r2 | 3 |  | Labrador City NL |
| NL.2012.0280 | r1 | 3 | 17 | Labrador City NL |
| NL.2012.0322 | r2 | 3 | 18 | Labrador City NL |
| NT.2013.0108 | r13 |  |  | Paulatuk NT |
| MB.2012.0785 | r3 | 1 |  | Churchill MB |
| MB.2012.0888 | r3 | 1 | 17 | Churchill MB |
| NU.2012.0085 | r12 | 1 |  | Kuugaruk NU |
|  |  |  |  |  |
| **Arctic Foxes** (n=27) |  |  |  |  |
| NT.2012.1020 | a14 |  | 18 | Holman NT |
| NU.2012.0113 | a1 |  |  | Rankin Inlet NU |
| NU.2012.0150 | a4 |  | 18 | Kuugaruk NU |
| NU.2012.0176 | a4 |  |  | Kuugaruk NU |
| NT.2012.0020 | a3 |  |  | Sachs Harbour NT |
| NU.2012.0207 | a9 |  | 18 | Arviat NU |
| NU.2012.0260 | a1 |  | 2 | Cambridge Bay NU |
| NT.2012.0042 | a4 |  | 2 | Sachs Harbour NT |
| NT.2012.0043 | a1 |  |  | Sachs Harbour NT |
| NU.2012.0031 | a1 |  |  | Gjoa Haven NU |
| NU.2012.0988 | a1 |  | 17 | Cambridge Bay NU |
| NT.2012.0989 | a30 |  | 2 | Holman NT |
| NT.2013.0098 | a1 |  |  | Sachs Harbour NT |
| NT.2013.0040 | a1 |  | 18 | Holman NT |
| NT.2013.0087 | a12 |  |  | Sachs Harbour NT |
| NT.2013.0088 | a29 |  | 18 | Sachs Harbour NT |
| NT.2013.0089 | a1 |  |  | Sachs Harbour NT |
| NT.2013.0094 | a7 |  | 8 | Paulatuk NT |
| QC.2012.0133 | a1 |  | 17 | Umiujaq QC |
| QC.2012.0243 | a15 |  | 17 | Umiujaq QC |
| QC.2012.0378 | a2 |  | 17 | Puvirnituq QC |
| QC.2012.0379 | a7 |  | 17 | Ivujivik QC |
| QC.2012.0528 | a5 |  | 18 | Salluit QC |
| NU.2013.0304 | a3 |  | 18 | Igloolik NU |
| NU.2013.0305 | a3 |  | 2 | Igloolik NU |
| NU.2013.0473 | a6 |  | 18 | Resolute Bay NU |
| NU.2013.0643 | a1 |  | 2 | Grise Fiord NU |
