# Supplementary material for: Relationships between fox populations and rabies virus spread in northern Canada
Source: PLoS One. 2021 Feb 16;16(2):e0246508. doi: 10.1371/journal.pone.0246508 (PMC7886166; doi:10.1371/journal.pone.0246508)
Supplement: Attachment — Submitted filename: Respone to reviewers.docx [file pone.0246508.s009.docx]

Re: PONE-D-20-18736
Relationships between fox populations and rabies virus spread in northern Canada
PLOS ONE

All comments as listed below have been addressed with each response clearly identified. Note that line numbers refer to the clean copy version.

Journal Requirements:

When submitting your revision, we need you to address these additional requirements.

1. Please ensure that your manuscript meets PLOS ONE's style requirements, including those for file naming. The PLOS ONE style templates can be found at

<https://journals.plos.org/plosone/s/file?id=wjVg/PLOSOne_formatting_sample_main_body.pdf> and

<https://journals.plos.org/plosone/s/file?id=ba62/PLOSOne_formatting_sample_title_authors_affiliations.pdf>

**Response**: The manuscript has been revised according to the guidelines.

2. We note that you are reporting an analysis of a microarray, next-generation sequencing, or deep sequencing data set. PLOS requires that authors comply with field-specific standards for preparation, recording, and deposition of data in repositories appropriate to their field. Please upload these data to a stable, public repository (such as ArrayExpress, Gene Expression Omnibus (GEO), DNA Data Bank of Japan (DDBJ), NCBI GenBank, NCBI Sequence Read Archive, or EMBL Nucleotide Sequence Database (ENA)). In your revised cover letter, please provide the relevant accession numbers that may be used to access these data. For a full list of recommended repositories, see http://journals.plos.org/plosone/s/data-availability#loc-omics or <http://journals.plos.org/plosone/s/data-availability#loc-sequencing>

**Response**: The viral sequence data and new fox control region haplotype sequences generated during this study have been deposited to GenBank and the Accession numbers are provided in the data availability statement.

3. We note that [Figures 1, 4 and S1] in your submission contain [map/satellite] images which may be copyrighted. All PLOS content is published under the Creative Commons Attribution License (CC BY 4.0), which means that the manuscript, images, and Supporting Information files will be freely available online, and any third party is permitted to access, download, copy, distribute, and use these materials in any way, even commercially, with proper attribution. For these reasons, we cannot publish previously copyrighted maps or satellite images created using proprietary data, such as Google software (Google Maps, Street View, and Earth). For more information, see our copyright guidelines: <http://journals.plos.org/plosone/s/licenses-and-copyright>.

We require you to either (1) present written permission from the copyright holder to publish these figures specifically under the CC BY 4.0 license, or (2) remove the figures from your submission:

The following resources for replacing copyrighted map figures may be helpful:

Natural Earth (public domain): http://www.naturalearthdata.com/

**Response**: All maps in Figs 1, 4 and S1 have been remade using materials from the Natural Earth website. In addition, Figs 4 and S1 illustrate provincial and territorial boundaries using a freely available shapefile from Statistics Canada, acknowledged as per terms of use (see additional documentation).

Reviewers' comments:
5. Review Comments to the Author

Reviewer #1: This manuscript provides new data and analysis on rabies virus and host genetics in the Canadian North
The paper is very well written and the experiments and analysis are very well described and appropriate to address the question of rabies spread and maintenance in the Canadian North. Conclusions are well supported by the data presented. The findings and discussion contribute to our understanding of rabies dynamics is the far North, and analyses virus spread into more southern regions.
I only have a few minor comments:

Line 89: should this be last century instead of earlier this century? How much has fox farming decreased in the last 20 years vs 50 or 100- years?

**Response:** Yes. This has been changed to last century (line 87). Fox farming has been quite limited in the area. In Labrador one fox farm operated in the Cartwright area for a short time in the 1910s as mentioned in the text and a farm operated in the Happy Valley-Goose Bay area a little to the west of Cartwright in the 1980s and 1990s but no fox farms are currently operational. Apart from the Cartwright situation as described we do not feel that fox farming has had a significant impact across the rest of northern Canada.

Line 701: sentence needs to be corrected

**Response:** The redundant “has” was removed (now line 751).

Line 708: Should this be group A3- 4 instead of group 4?

**Response:** For consistency throughout the document, all A3 groups have been designated as A3-X where X refers to the specific group number.

Reviewer #2: This is an interesting study and an impressive amount of work and sampling considering the remote locations being studied. However, I think the presentation could be improved. In particular, more details are needed in the methods, and some of the most interesting findings are presented in text only and are not visible on the figures.

Major comments:

Most of the conclusions about how rabies spreads within and between hosts cannot currently be verified, because host information is missing from key figures. Please annotate the time-scaled phylogeny and the maps with at least the major hosts (e.g. arctic fox, red fox, other).

Line 119: More detail about temporal differences in surveillance is needed, as the data covers a very long time period. I was left wondering for how much of this period non-contact animals had been tested, and how such samples were treated before the DRIT became available. I was under the impression that the DRIT is a relatively recent development?

**Response:** The reviewer brings up a very salient point that surveillance in northern parts of Canada has undergone some significant changes over the last 40+ years. At the federal level restrictions were imposed such that only human/domestic animal contacts were accepted for testing. Provincial authorities have used the DRIT to try to expand the scope of surveillance by testing additional cases. Social changes have also played a significant role. We have addressed these issues by adding the date of DRIT adoption by provincial labs into the methods section (lines 115-118). We have also added several comments on the changing surveillance landscape in the discussion (lines 785-789).

Line 148-150: More details needed here:
1. Which alignment algorithm was used?
2. Which substitution models were used for these analyses?
3. Three distinct phylogenetic methods are discussed, but the maximum likelihood results are never presented. I don’t think all three are necessarily needed, but arguably the maximum likelihood phylogeny would be more robust than the neighbour joining analysis presented, if both are available.

**Response:** A rewrite of this section (lines 147-161) has added the following:

1. The use of the MUSCLE alignment program,

2. Use of the Tamura-3 parameter substitution model for N gene analysis

3. Use of the GTR+G+I model for analysis of the whole genome sequencing dataset.

With respect to the phylogeny illustrated in Fig 2 the neighbor joining tree has been replaced with the maximum likelihood tree as suggested. Due to a small difference in the presentation of samples from Ontario this required alteration of the text (lines 288-298).

Line 162: Which phylogeny was used in these analyses? The preceding text describes multiple phylogenetic reconstructions, and here a new alignment is introduced. Since nothing is specified I’d assume the authors relied on automatic reconstructions of phylogeny by HyPhy/datamonkey, but if so this needs to be specified, ideally along with the substitution model used in these instances. Using the same phylogeny as presented elsewhere would be better, but otherwise at least a statement confirming that the topology didn’t differ from what is shown is needed.

**Response:** Automatic phylogeny reconstruction by the HyPhy/datamonkey methods was employed. Using the GTR substitution model the phylogenies thus constructed were similar to those that are presented in the figures. The text has been modified accordingly (lines 179-180).

Line 237: Which diversity measures were used? A very large number of measures exists (e.g. Shannon, Simpson, etc.), and a quick look at the Arlequin documentation suggests it can calculate several (though the documentation does not specify which).

**Response:** The measures that were used were as listed in brackets. These are specific diversity measures in and of themselves. To better clarify this the sentence has been rephrased (lines 255-258).

Line 269: Clade A1 is not strongly supported – the node grouping all A1 sequences has just 51% bootstrap support, and no value is shown for the split between A1 and all other clades.

**Response:** As indicated above the NJ tree has been replaced by the ML tree. Interestingly the ML phylogeny supports separation of the A1 clade into two separate groups as the revised text describes (lines 288-298) although the time-scaled tree does support the monophyletic nature of the A1 clade as well as its division into two discrete groups.

Line 282: The methods did not contain information about how the outgroup was chosen. Also, more information should be given here:
1. Please annotate major hosts (as discussed above)
2. Which of the tips represent sequences generated in this study?
3. Do clusters correspond to anything (e.g. broad locations)?

Line 319: As above, this figure should show hosts, and here knowing the hosts becomes central to the arguments of the paper.

**Response:**

1. The N gene phylogeny presented in Fig 2 has been revised to include symbols indicating the host species for each sample. As now detailed in the Methods section (lines 155-158) the outgroup sequence used in this tree belongs to the rabies virus Arctic-like lineage which is the most closely related to the true Arctic lineage. We felt that a member of this lineage was the most appropriate as an outgroup.

2. A footnote added to S1 Table identifies the sequences generated during the study.

3. The addition of host information clearly demonstrates that, except for the A1 samples that were recovered outside the range of the Arctic fox, the viral clusters do not generally correspond to differences in reservoir host. The geographical distribution of the A3 groups is better illustrated by the maps in Figure 4 which now include host information (see below).

Line 324: “posterior value” is vague – presumably this should be “posterior probability”?

**Response:** Yes, this has been altered (line 354).

Line 327: Can the cases with no precise coordinates be illustrated another way, e.g. by writing the number of cases in the centre of Alaska, with the font coloured the same as the group? At present it’s unclear from the maps and other figures where Alaskan samples fit in and whether they are all a single phylogenetic group.

**Response:** Additional text now better describes our findings for the state of Alaska (lines 298-303 and 312-320). Cases representing three sub-lineages, A2-A4, were recovered from the state and they exhibited some geographical localisation as noted in prior studies. As the maps presented in Figure 4 illustrate only viruses of the A3 sub-lineage, this being the only sub-lineage currently circulating across Canada, and only one A3 virus case from Alaska could be mapped, it was decided to remove the state from these maps.

Line 350: Point shapes should reflect sampled host. Potential changes in host as the virus spreads are discussed in the text, so it would be nice to be able to see this and to get a sense of how many cases are being used to support such statements.

**Response:** The revised maps presented in Figure 4 now include host data using different point shapes as detailed in the figure legend.

Line 373: When giving estimated dates of MRCAs, please include some measure of confidence, such as a 95% credible interval / HPD.

**Response:** A 95% HPD range has been added to all date estimates (lines 337-347).

Line 615-618: I could not follow this argument – how can the suggested limited level differentiation be supported by findings of significant differentiation? The following sentence goes on to say that others have found North/South differentiation.

**Response:** We agree that this statement is somewhat confusing in the present context so this sentence and the following one have been deleted. The following paragraph has also been moved to provide better flow to the narrative. Reference to the proposed north-south division of the Arctic fox population has also been removed from the abstract given that the evidence for this is tenuous.

Minor comments:

Line 35-38: It took me a while to understand this sentence and that it is the species which differ, and not the data types giving conflicting results. This might be clearer with an added “both”, as in: “…, but both mitochondrial DNA control region sequences and 9-locus microsatellite genotypes revealed…”

**Response:** The sentence has been modified by adding “both” as suggested (line 34).

Line 55: Technically this should read “Rabies lyssavirus (RABV)…”, as the sentence is referring to the species.

**Response:** The sentence has been modified as suggested (line 53).

Line 134: The acronym “AFX” is used throughout the manuscript but has not been defined as far as I can tell. Since only one variant is being discussed and there are many other acronyms to remember, is this one needed?

**Response:** The AFX acronym has been replaced with “Arctic lineage” throughout.

Line 194: In figure 1, it is very hard to distinguish the small circles from small squares used to plot “other” locations.

**Response:** Fig 1 has been replaced as detailed above and the points better distinguished.

Line 199: Potential typo: should this be “[hamstermap.com](http://hamstermap.com/)”?

**Response:**  This has been removed from the figure legend due to the map revision.

Line 283: The methods said version 7 of MEGA was used.

**Response:** Yes MEGA 7 was used and this has been adjusted (line 306).

Line 372: Unclear why the numbering scheme changes for these lineages, both here and in the plot. Earlier A3 sub-lineages were simply referred to by number.

**Response:** To avoid any confusion between A3 groups and the other sub-lineages, all A3 group members are now designated by A3-X as indicated above.

Line 511: Is this line meant to refer to table S5 instead?

**Response:** Yes. This has been corrected (line 563).

Line 589-590: I found the final part of this sentence confusing, since no results contrasting the rabies and host population genetics results had been presented by this point. Perhaps write: “allowing us correlate…” or “this allows us to correlate”, to indicate that this is still coming in the next paragraphs.

**Response:** This has been revised as suggested (line 642).

Line 736: Is the word “detection” missing here?

**Response:** The word “detection” has been added (line 792).

Supplementary document:
1. Caption for table S3 is incomplete, with placeholder text where the authors intended to add accession numbers.

**Response:** This text has been removed since the Accession numbers are provided in the data availability statement.

2. Caption for table S4 refers to a “Table d1”, which is presumably a typo.

**Response:** Typo has been corrected by removal of “d”.
